# Inflammaging-induced TRAF3 degradation impairs AMP biosynthesis to drive sarcopenia

**DOI:** 10.21203/rs.3.rs-8857997/v1

**Published:** 2026-03-20

**Authors:** Yaning Xing, Jinxiao Fan, Xing Li, Tian Jin, Congcong Zhang, Lilong Dong, Xiaokuan Zhang, Zhihui Liu, Pinliang Li, Qingfeng Yang, Tao Wu, Brendan Boyce, Jinbo Li

**Affiliations:** 1 Department of Pharmacology, Hebei Medical University, Shijiazhuang, China; 2 Department of Pathology, University of Rochester Medical Center, Rochester, NY, United States; 3 The Key Laboratory of Neural and Vascular Biology, Ministry of Education of China, Hebei Medical University, Shijiazhuang, China; 4 Hebei Key Laboratory of Cardiovascular Homeostasis and Aging, Hebei Medical University, Shijiazhuang, China; 5 Department of Cancer Immunotherapy, the Fourth Hospital of Hebei Medical University, Shijiazhuang, China; 6 Department of Medical and Pharmaceutical Informatics, Hebei Medical University, Shijiazhuang 050017, China; 7 School of Nursing, Hebei Medical University, Shijiazhuang, Hebei, 050017, China; 8 Department of Bone Disease, Third Hospital of Hebei Medical University, Shijiazhuang, China

**Keywords:** inflammaging, sarcopenia, TRAF3, TGFβ1, neutrophil, ADSL, AMP

## Abstract

Inflammaging is a recognized driver of age-related pathologies, yet its specific mechanistic link to sarcopenia remains poorly understood. Here, we identified a significant reduction of TNF receptor-associated factor 3 (TRAF3) in myoblasts exposed to aged serum and in skeletal muscles from both aging mice and humans. Genetic deletion of TRAF3 in myocytes or satellite cells induced early-onset sarcopenia and impaired regeneration, independent of non-canonical NF-κB signaling. Mechanistically, TRAF3 maintains energy homeostasis by stabilizing the key metabolic enzyme, adenylosuccinate lyase (ADSL), and its loss impairs AMP biosynthesis and ATP production. Muscle-specific TRAF3 restoration or AMP supplementation rescued sarcopenic phenotypes in TRAF3-deficient mice. Notably, neutrophil-derived transforming growth factor β1 (TGFβ1) caused IAP-mediated ubiquitination and degradation of TRAF3 in aged mice—a process reversible by the IAP inhibitor SM-164. Inducible neutrophil-specific TGFβ1 deletion prevented age-related sarcopenia. Our study establishes that TRAF3 is a key protective factor in muscle aging, and its loss mechanistically links inflammaging to bioenergetic deficits, suggesting new strategies to prevent age-related muscle wasting.

## Introduction

Age-related sarcopenia is a debilitating condition characterized by the progressive loss of skeletal muscle mass and function^1^, profoundly impacting the health and quality of life of older adults. It affects approximately 10% of individuals aged 60–70 years and up to 50% of those over 80 years^2^. This decline in muscle health leads to impaired mobility^3^, increased risk of falls and fractures^4^, and compromised capacity for muscle repair^5^. The etiology of sarcopenia is multifactorial, arising from a complex interplay of neuromuscular and hormonal changes, anabolic resistance^6^, mitochondrial dysfunction^7^, and notably, low-grade chronic inflammation (LLCI)^8^.

LLCI, often termed “inflammaging”, is increasingly recognized as a key contributor to a spectrum of age-related pathologies^9^, including Alzheimer’s diseases^10^, diabetes^11^, osteoporosis^12^ and sarcopenia^8^. In aged skeletal muscle, LLCI is marked by elevated levels of pro-inflammatory cytokines, such as TNFα^13^, alongside infiltration by mainly myeloid-lineage cells, particularly macrophages^14^ and neutrophils^15^. Macrophage-derived TNFα has been shown to promote skeletal muscle fibrosis, myosin heavy chain (MyHC) degradation, and satellite cell dysfunction^16, 17^. Neutrophils, while crucial for initiating the inflammatory phase of muscle repair, can cause significant collateral damage to healthy tissue upon excessive or prolonged infiltration^18, 19^. We^20^ and others^21^ have reported a significant accumulation of neutrophils in skeletal muscle during aging, yet their precise role and secretory profile in driving age-related sarcopenia remain largely uncharacterized.

The signaling pathways that mediate the effects of inflammatory cues in muscle are central to this process. TNF receptor-associated factor (TRAF) proteins, comprising TRAF1 to TRAF7, are a family of crucial intracellular adaptors that function as signaling hubs for a wide range of receptors, including TNF receptors, Toll-like receptors (TLRs), and interleukin-1 receptors^22^. For example, in response to TNFα, TRAF6 stimulates expression of the muscle-specific E3 ligases, MuRF-1 and Atrogin-1, exacerbating muscle atrophy in sarcopenia models^20^. TRAF4 interacts with the AKT signaling pathway, thereby promoting muscle fibrosis during atrophy^23^. In contrast, TRAF3 often functions as a negative regulator of non-canonical NF-κB signaling by promoting the degradation of NF-κB-inducing kinase (NIK)^24–26^. Specific deletion of TRAF3 from mesenchymal lineage cells results in early-onset osteoporosis in mice due to enhanced β-catenin degradation^27^, underscoring its importance in tissue homeostasis during aging. However, the roles of TRAF proteins, particularly TRAF3, in response to LLCI within the context of age-related sarcopenia are still poorly defined.

Skeletal muscle is not merely a contractile organ, but also a major metabolic hub, constituting over 40% of adult body mass and serving as the primary site for glucose disposal^28^, fatty acid oxidation^29^, amino acid reserves, and energy expenditure^30^. It consumes over 90% of the body’s energy during intense exercise, a massive energy demand met by self-generated ATP, the sole immediate energy source for contraction^31^. Aging is linked to significant defects in muscle ATP generation, which is increasingly viewed as a primary driver of sarcopenia^32^. While mitochondrial dysfunction contributes to this energy deficit^33^, it remains unclear how the age-associated inflammation landscape mechanistically disrupts these critical metabolic processes to impair energy supply.

Thus, while chronic inflammation is an established driver of age-related sarcopenia, the precise molecular mechanisms that translate inflammatory signals into the metabolic and functional decline of muscle fibers remain elusive. A critical unanswered question is how the inflammatory milieu of aged muscle directly impairs cellular energy supply. Here, we identify the degradation of TRAF3 as a key mechanism linking ‘inflammaging’ to sarcopenia. We demonstrate that neutrophil-derived TGFβ1 promotes TRAF3 loss, which in turn disrupts AMP biosynthesis by failing to stabilize the metabolic enzyme, ADSL, thereby creating a critical energy deficit. Our findings establish a novel inflammation-metabolism axis in sarcopenia and unveil potential therapeutic strategies for age-related muscle wasting.

## Results

### TRAF3 protein levels are reduced in elderly human and mouse skeletal muscles

**Figure 1.**
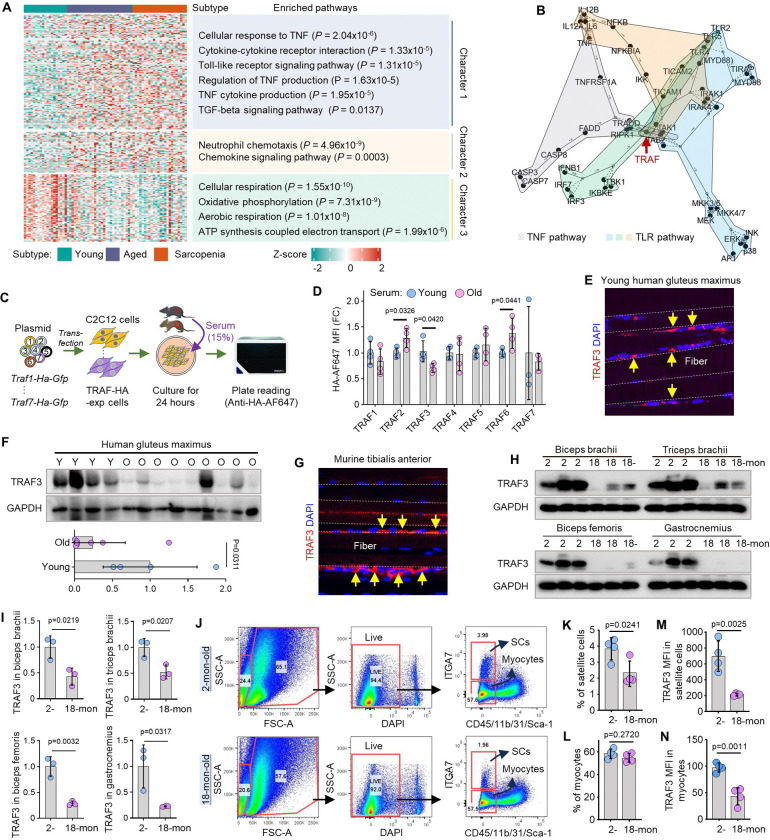
TRAF3 protein levels are reduced in elderly human and mouse skeletal muscles. (A) Summary of transcriptomic analysis of human skeletal muscle, primarily highlighting upregulation of chronic inflammation and downregulation of mitochondrial metabolism gene sets in specimens from elderly individuals, with or without sarcopenia, compared to young controls. (B) Schematic illustrating the convergence of TNF and Toll-like receptor (TLR) signaling pathways on TNF receptor-associated factor (TRAF) proteins. (C-D) Experimental design of mouse myoblasts transfected with HA-tagged TRAF plasmids, and conditionally cultured with 15% mouse serum from young (Y; 2 mon-old) and old (O; 20 mon-old) mice for measurement of the levels of HA-tagged TRAFs. (E) Representative images showing TRAF3 protein expression (yellow arrows) in a human gluteus maximus specimen by IF. (F) TRAF3 and GAPDH protein levels in gluteus maximus tested by WB. (G) IF staining for TRAF3 protein expression (yellow arrows) in mouse tibialis anterior (TA). (H) TRAF3 and GAPDH protein levels in biceps brachii, triceps brachii, biceps femoris, and gastrocnemius muscles tested by WB. (I) Quantification of TRAF3 protein levels normalized to GAPDH levels in various skeletal muscles in (H). (J) Flow gating strategies reveal myocytes (CD45^−^CD11b^−^CD31^−^Sca1^−^ITGA7^−^) and satellite cells (CD45^−^CD11b^−^CD31^−^Sca1^−^ITGA7^+^) in live (DAPI^−^) singlets digested from skeletal muscles from 2- and 18-mon-old mice. (K-L) The frequencies of satellite cells and myocytes in digested muscle cells from 2- and 18-mon-old mice. (M-N) Mean fluorescence intensity (MFI) of TRAF3 expression by satellite cells and myocytes. All data are presented as mean ± SD. Analysis: Student’s unpaired *t* test.

Transcriptomic analysis of human skeletal muscle revealed an age-associated inflammatory signature, characterized by the concerted upregulation of TNF, TLR, and TGFβ signaling pathways (Character 1), as well as chemokine-mediated neutrophil infiltration (Character 2) (Fig. 1A). As these inflammatory pathways converge on TRAF family proteins (Fig. 1B), we investigated their potential role in age-related sarcopenia.

Transcriptomic analysis of two independent cohorts of human skeletal muscle demonstrated that mRNA levels of *Traf1-Traf7* were unchanged in human skeletal muscle during aging (Suppl. Fig. 1A-B), while exposure of *Traf1–7*-HA-transfected C2C12 myoblasts to serum from aged mice significantly reduced TRAF3 protein levels, contrasting with increased TRAF2 and TRAF6 levels (Fig. 1C–D). TRAF3 degradation is promoted by TRAF2-mediated ubiquitination, but its role in age-related sarcopenia has not yet been established.

Notably, TRAF3 protein was highly expressed in myocytes in gluteus maximus muscle from young human donors (Fig 1E), and its expression was significantly lower in skeletal muscle specimens from older human donors compared to young controls (Fig. 1E–F). This age-related decline was recapitulated in samples from multiple muscles of old mice, including the tibialis anterior (TA), biceps, triceps brachii, biceps femoris, and gastrocnemius muscles (Fig. 1G–I). Flow cytometric analysis of muscle-derived cells showed that this TRAF3 reduction occurred within both myocytes (CD45^−^CD31^−^CD11b^−^Sca1^−^ITGA7^−^) and satellite cells (CD45^−^CD31^−^CD11b^−^Sca1^−^ITGA7^+^), the latter also being less frequent in old muscles (Fig. 1J–N).

### Mice with TRAF3 specific deletion in myocytes or satellite cells develop early-onset sarcopenia

**Figure 2.**
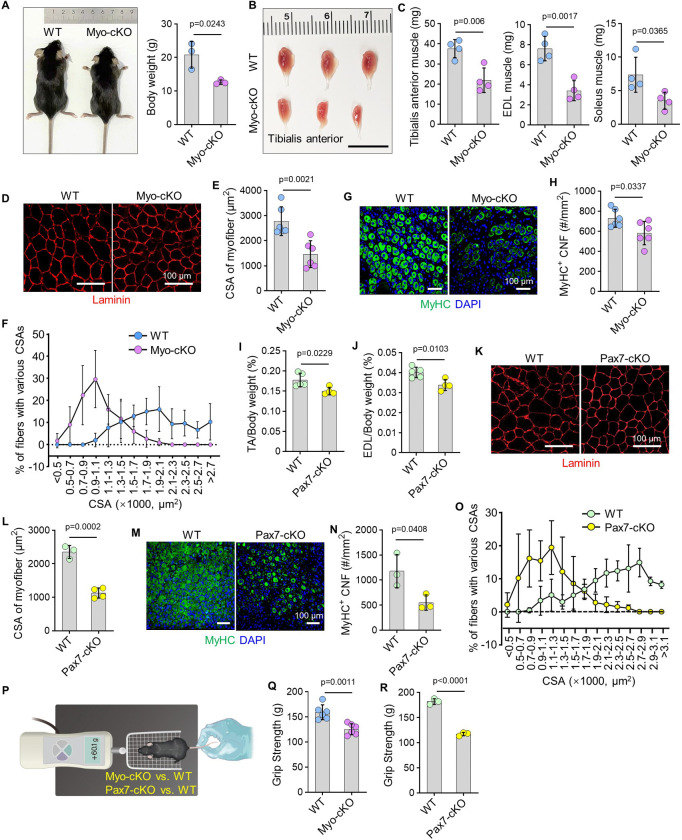
Mice with TRAF3 specific deletion in myocytes or satellite cells develop early-onset sarcopenia. (A) Representative images of 3-mon-old MCK^Cre^TRAF3^fl/fl^ (refers to Myo-cKO) and TRAF3^fl/fl^ (WT) littermates, and their body weights. n=3 mice/group. (B) Representative images of tibialis anterior (TA) muscles from 3-mon-old Myo-cKO and WT mice, and (C) muscle mass of TA, extensor digitorum longus (EDL) and soleus muscles. n=4 mice per group. (D) IF staining for laminin expression on cryosections of TA muscles from 3-mon-old Myo-cKO and WT mice, and (E) cross sectional area (CSA) of TA myofibers. n=6 mice/group. (F) Myofibers with cross-sectional areas in TA muscles from 3-mon-old Myo-cKO and WT mice. (G) IF staining for MyHC expression by regenerated centrally nucleated fibers (CNFs) on cryosections of injured TA muscles 5 d after barium chloride (BaCl_2_) injection, and (H) the number of MyHC^+^ myocytes with CNFs. n=6 mice per group. (I-J) Weights of TA and EDL muscles, normalized to their respective body weights, from 4-mon-old Pax7^CreER^TRAF3^fl/fl^ (refers to Pax7-cKO) mice and TRAF3^fl/fl^ (refers to WT) mice. n=5 WT and 4 Pax7-cKO mice. (K) IF staining for laminin expression in cryosections of TA muscles from 4-mon-old Pax7-cKO and WT mice, and (L) CSA of TA myofibers. n=3 WT and 4 Pax7-cKO mice. (M) IF staining for MyHC expression by regenerated CNFs in cryosections of injured TA muscles 5 d after BaCl_2_ injection, and (N) number of MyHC^+^ myocytes with CNFs. n=3 mice/group. (O) TA myofibers with CSAs from 4-mon-old Pax7-cKO and WT mice. (P-R) Grip strength of Myo-cKO and Pax7-cKO mice compared to their respective WT control mice. n=6 mice for Myo-cKO and WT mice, 3 mice for Pax7-cKO and WT mice. Analysis: Student’s unpaired *t* test.

To determine if TRAF3 loss in myocytes causes skeletal muscle atrophy, we generated myocyte-specific TRAF3 conditional knockout (MCK^Cre^TRAF3^fl/fl^; Myo-cKO) mice. At 3 months of age, Myo-cKO mice had a pronounced sarcopenic phenotype, characterized by significantly reduced body weight (Fig. 2A), decreased mass of the TA, extensor digitorum longus (EDL), soleus, gastrocnemius, and quadriceps muscles (Fig. 2B–C), reduced myofiber cross-sectional area (CSA) (Fig. 2D–F), and a marked loss of nuclei per myofiber (Suppl. Fig. 1C-D), with no gender difference (Suppl. Fig. 1A-B). The observed sarcopenic phenotype in cKO mice occurred without alterations in the proportions of satellite cells (Pax7^+^) or mature myocytes (MyoD^+^), as shown in Suppl. Fig. 1E-F. *In vitro*, magnetically sorted myoblasts from Myo-cKO mice formed significantly fewer myofibers than those from wild-type (WT) controls (Suppl. Fig. 1G-H). Consistent with this, following barium chloride-induced injury, Myo-cKO muscles exhibited impaired regenerative capacity, as evidenced by fewer and smaller newly regenerated fibers with central nuclei (CNFs) (Fig. 2G–H; Suppl. Fig. 1I-J), accompanied by a significant expansion of the inflammatory area within injured muscle (Suppl. Fig. 1K-L).

To investigate the contribution of TRAF3 loss in satellite cells, we induced TRAF3 deletion in Pax7-expressing satellite cells of Pax7^CreER^TRAF3^fl/fl^ (Pax7-cKO) mice and their wild-type (WT) littermates through tamoxifen administration at 15, 75 and 105 days of age (Suppl. Fig. 2A). Mirroring the Myo-cKO phenotype, Pax7-cKO mice showed significant muscle atrophy, with lower normalized mass of the TA, EDL, gastrocnemius, and quadriceps muscles (Fig. 2I–J; Suppl. Fig. 2B-C), and reduced myofiber CSA (Fig. 2K–L, 2O). Body weight and the composition of satellite cells (Pax7^+^) and mature myocytes (MyoD^+^) remained unchanged (Suppl. Fig. 2D-F). In addition, Pax7-cKO mice also had impaired regenerative capacity post-injury, forming fewer and smaller newly regenerated CNFs (Fig. 2M–N; Suppl. Fig. 2G-H), and showing a significant increase in the inflammatory area within injured muscle (Suppl. Fig. 2I-J).

Notably, the muscle wasting culminated in a significant reduction in grip strength in both Myo-cKO and Pax7-cKO mice relative to their respective WT controls (Fig. 2P–R). Collectively, these data demonstrate that specific deletion of TRAF3 in either myocytes or satellite cells is sufficient to drive early-onset sarcopenia in mice.

### Sarcopenia in Myo-cKO mice with TRAF3 deficiency is NF-κB-independent

**Figure 3.**
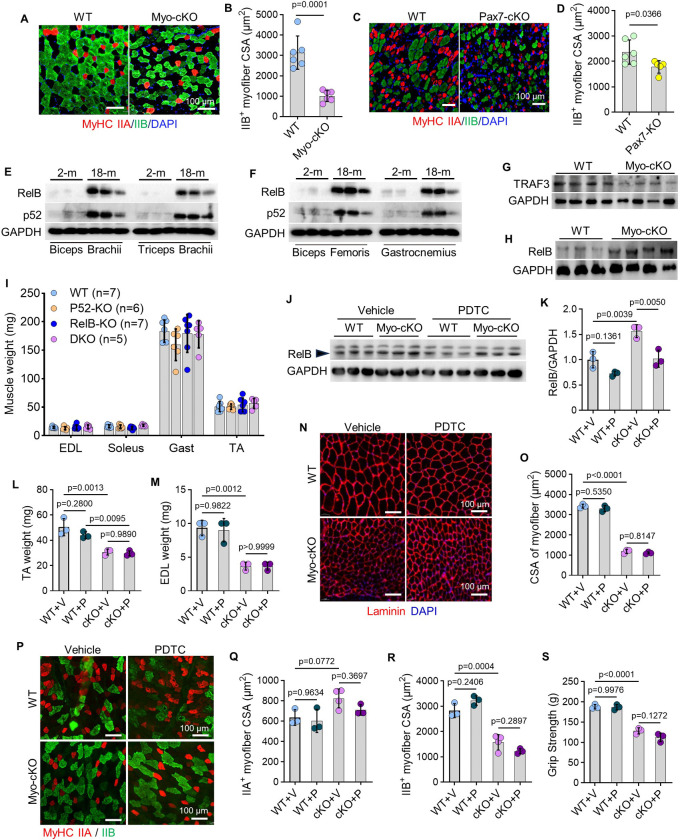
Sarcopenia in Myo-cKO mice with TRAF3 deficiency is NF-κB-independent. (A) Representative images showing IF staining for MyHC IIA (red) and IIB (green) in cryosections of TA muscles from 3-mon-old Myo-cKO and WT mice, and (B) CSAs of type IIB fibers. n=6 mice per group. (C) IF staining for MyHC IIA (red) and IIB (green) in cryosections of TA muscles from 4-mon-old Pax7-cKO and WT mice, and (D) CSAs of type IIB fibers. n=6 WT and 5 Pax7-cKO mice. (E-F) WBs of RelB, p52 and GAPDH levels in various muscles from young and old mice. n=3 mice/group. (G-H) WBs of TRAF3 and RelB expression in gastrocnemius muscles from Myo-cKO and WT mice. n=3–4 mice/group. (I) Weights of TA, EDL, soleus, and gastrocnemius muscles from p52 and RelB single- and double-knockout mice and WT mice. (J) WBs of RelB and GAPDH levels in gastrocnemius muscles from Myo-cKO and WT mice treated with the NF-κB inhibitor, PDTC, or vehicle. n=3 mice per group. (K) RelB protein levels normalized to GAPDH in each group shown in (J). n=3 mice/group. (L-M) Weights of TA and EDL muscles from Myo-cKO and WT mice treated with PDTC and vehicle. n=3 mice/group. (N) IF staining for laminin expression on cryosections of TA muscles from Myo-cKO and WT mice treated with PDTC or vehicle, and (O) CSA of TA myofibers. n=3 mice/group. (P) IF staining for MyHC IIA (red) and IIB (green) on cryosections of TA muscles from vehicle- and PDTC-treated Myo-cKO and WT mice, and (Q-R) CSAs of type IIA and IIB fibers. n=4 vehicle-treated Myo-cKO mice and 3 mice for other groups. (S) Grip strength of vehicle- and PDTC-treated Myo-cKO and WT mice. n=3 mice/group. Analysis: Student’s unpaired *t* test in (B) and (D); one-way ANOVA with Tukey post analysis for all others.

We next investigated the mechanistic basis of sarcopenia in TRAF3-deficient mice. Histological analysis revealed a common pathological feature in both Myo-cKO and Pax7-cKO muscles: a significant reduction in the CSA of fast-twitch, type IIB myofibers compared to their respective WT controls (Fig. 3A–D). In addition, Myo-cKO and Pax7-cKO muscles had a significant loss in the number and size of fast-twitch, type IIA myofibers compared to WT controls, respectively (Suppl. Fig. 3A-D). Given that TRAF3 is a well-established negative regulator of the non-canonical NF-κB pathway^43^, we assessed its activity. Concordant with the age-associated decline in TRAF3 (Fig. 1D–F), protein levels of the non-canonical NF-κB components, p52 and RelB, were increased in skeletal muscles from aged mice (Fig. 3E–F; Suppl. Fig. 3E-H). Furthermore, gastrocnemius muscles from TRAF3-deficient Myo-cKO mice had a concomitant increase in RelB protein levels (Fig. 3G–H; Suppl. Fig. 3I-J).

A series of experiments, however, demonstrated that this pathway does not primarily drive the sarcopenic phenotype. First, global genetic ablation of *p52* and *RelB*—individually or in combination—failed to increase the mass of the TA, EDL, soleus, or gastrocnemius muscles compared to WT mice (Fig. 3I). Although the satellite cell population was reduced in p52/RelB-double knockout (DKO) muscles (Suppl. Fig. 3K), these data indicate that muscle mass is not directly governed by non-canonical NF-κB signaling in this context.

We next employed a pharmacological approach to directly inhibit NF-κB. Treatment of Myo-cKO and WT mice with ammonium pyrrolidine dithiocarbamate (PDTC) (Suppl. Fig. 3L), a selective inhibitor that blocks IκB phosphorylation and NF-κB nuclear translocation, effectively reduced RelB protein levels in skeletal muscles (Fig. 3J–K). Despite this successful inhibition, PDTC treatment did not rescue the sarcopenic phenotype in Myo-cKO mice. There was no significant improvement in body weight, muscle mass (TA, EDL, soleus, gastrocnemius) (Fig. 3L–M; Suppl. Fig. 3M-Q), overall myofiber CSA, or the specific atrophy of type IIA/IIB myofibers (Fig. 3N–R). The reduced grip strength also remained unchanged (Fig. 3S). Taken together, these genetic and pharmacological findings establish that the heightened non-canonical NF-κB activity observed in TRAF3-deficient muscle is not a major contributor to the resultant sarcopenia.

### TRAF3 regulates AMP production by stabilizing ADSL tetramer and activity

**Figure 4.**
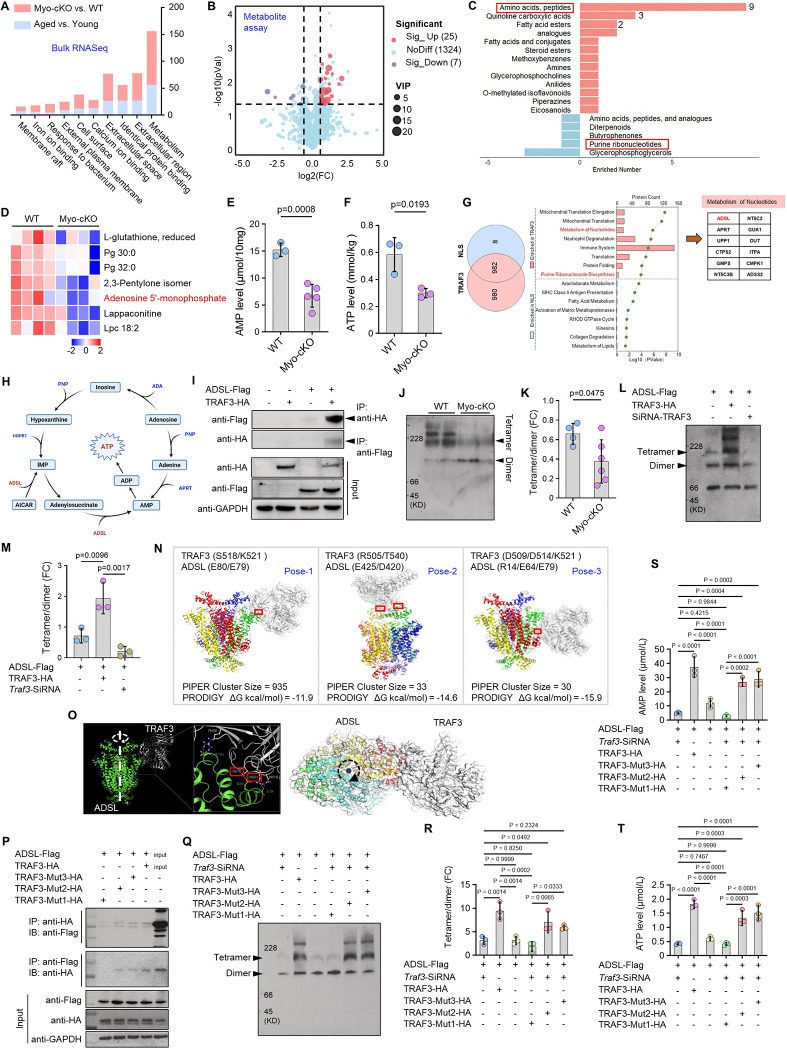
TRAF3 regulates AMP generation by stabilizing ADSL tetramer and activity. (A) Histogram showing significantly changed pathways in skeletal muscles from Myo-cKO mice and aged WT mice, compared to their respective controls. (B) Volcano graphs of significantly up-regulated (upper-right) and down-regulated (upper-left) metabolites detected in TA muscles from Myo-cKO mice compared to WT mice. (C) Major signaling pathways in which significantly changed metabolites are enriched, and (D) significantly down-regulated metabolites in TA muscles from Myo-cKO versus WT mice. (E-F) AMP and ATP levels in skeletal muscle lysates. n=3 WT and 5 Myo-cKO mice in (E), and 3 mice/group in (F). (G) Venn graph of detected protein binding to full-length or NLS-mutated TRAF3 tested by mass spectroscopy, and the signaling pathways of these enriched proteins, especially the proteins involved in purine ribonucleoside biosynthesis. (H) Sketch showing ADSL’s role in AMP and ATP biosynthesis. (I) C2C12 myoblasts transfected with TRAF3-HA and ADSL-Flag plasmids, anti-HA or -Flag Abs were applied in IP assays to confirm their association with each other. (J) Immunoblotting based on Native-PAGE of ADSL tetramer and dimer levels in skeletal muscles from WT and Myo-cKO mice. (K) Fold change (FC) of ADSL tetramer to dimer. n=4 WT and 6 Myo-cKO mice. (L-M) C2C12 myoblasts transfected with TRAF3-HA or *Traf3* siRNA, and anti-Flag Ab applied to detect ADSL dimer and tetramer levels in protein lysates. Fold change (FC) of ADSL tetramer to dimer. n=3 samples/group. (N) Integrated analysis of binding pose stability and affinity for the ADSL-TRAF3 complex. The 3 major binding modes were scored by correlating metrics of conformational dynamics (PIPER cluster size, indicating population stability) with predicted binding thermodynamics (PRODIGY ΔG, where more negative values indicate stronger affinity). (O) The side view (left) and top-down view (right) of the ADSL tetramer complex binding with TRAF3, calculated using Web Server Cluspro 2.0, and potential binding sites on TRAF3 (left). (P) C2C12 myoblasts transfected with ADSL-Flag and TRAF3-HA, or various mutated TRAF3-mut-HA, and the association between ADSL-Flag and various TRAF3 proteins tested using IP assays. (Q) C2C12 myoblasts treated with *Traf3* siRNA and subsequently transfected with ADSL-Flag and various TRAF3-HA plasmids, and measurement of ADSL dimer and tetramer levels by WB. (R) Fold change (FC) of ADSL tetramer to dimer. n=3 samples/group. (S-T) AMP and ATP levels in C2C12 myoblasts treated in (Q). n=3 samples/group. Analysis: Student’s unpaired *t* test in (E), (F) and (K), and one-way ANOVA with Tukey post analysis for all others.

To elucidate the mechanism driving TRAF3-deficient sarcopenia, we performed bulk mRNA sequencing on skeletal muscles from young (3-mon-old) and aged (22-mon-old) C57Bl6/J mice, as well as from young (3-mon-old) Myo-cKO and WT mice. Notably, the most significantly altered pathway in both Myo-cKO and aged muscle was “Metabolism” (Fig. 4A; Suppl. Fig. 4A-B), pointing to a metabolic etiology. Subsequent metabolomic profiling of Myo-cKO muscle identified 25 upregulated metabolites, primarily enriched in “amino acids, peptides”, and 7 downregulated metabolites compared to WT (Fig. 4B–C). This signature included a specific downregulation of the “purine ribonucleotides” pathway in Myo-cKO muscle, with adenosine 5’-monophosphate (AMP) levels being significantly reduced (Fig. 4C–D). This metabolite profile suggests that peptide accumulation may be secondary to an insufficient energy supply, as ATP is synthesized from AMP. Corroborating this, both AMP and ATP levels were significantly lower in Myo-cKO skeletal muscle (Fig. 4E–F).

To define TRAF3’s role in this metabolic defect, we performed mass spectrometry to identify its interacting partners in myoblasts. Proteins binding to full-length TRAF3 were enriched in pathways related to nucleotide metabolism and purine ribonucleotide biosynthesis, compared to those binding a TRAF3 mutant lacking a nuclear localization signal (NLS) (Fig. 4G; Suppl. Fig. 4C-D). Among these interactors was adenylosuccinate lyase (ADSL), the key enzyme that catalyzes the conversion of succinyladenosine monophosphate (SAMP) to AMP and fumarate in *de novo* purine synthesis (Fig. 4G–H). Direct physical interaction between TRAF3 and ADSL was confirmed by co-immunoprecipitation (Fig. 4I).

As the active cytosolic form of ADSL is a tetramer, we assessed its oligomeric state. In Myo-cKO muscle, ADSL tetramer levels were diminished, leading to a significantly reduced tetramer-to-dimer ratio compared to WT (Fig. 4J–K). In vitro, TRAF3 overexpression in C2C12 myoblasts promoted ADSL tetramer formation, whereas TRAF3 knockdown inhibited it (Fig. 4L–M), indicating that TRAF3 binding is necessary to maintain the active tetrameric formation of ADSL.

We next employed computational modeling to predict the TRAF3-ADSL binding interface critical for this stabilization. This analysis identified three potential interaction sites: Site1, TRAF3 residues S518/K521 binding to ADSL residues E80/E79; Site2, TRAF3 residues R505/T540 binding to ADSL residues E425/D420; and Site3, TRAF3 residues D514/D509/K521 binding to ADSL residues R14/E64/E79 (Fig. 4N–O). A combined assessment of conformational dynamics (PIPER cluster size) and binding thermodynamics (PRODIGY ΔG) pinpointed Pose-1 as the predominant binding conformation, characterized by the highest conformational stability and the most favorable predicted binding affinity (Fig. 4N–O). Co-immunoprecipitation assays revealed that only TRAF3-Mut1 mutant (S518A/K521A), designed to disrupt the predicted Pose-1 interface, failed to interact with ADSL (Fig. 4P). Consistent with this, in cells where *Traf3* was knocked down using a specific siRNA (with knockdown efficiency confirmed by immunoblotting; Suppl. Fig. 4E), reconstitution with TRAF3-Mut1 failed to rescue ADSL tetramer formation, in contrast to reconstitution with the other mutants or wild-type TRAF3 (Fig. 4Q–R). This loss-of-function was functionally consequential: AMP and ATP biosynthesis were significantly impaired only in cells reconstituted with the non-binding TRAF3-Mut1 mutant (Fig. 4S–T). These data establish that specific interaction via the Site1 interface is essential for TRAF3 to stabilize the active ADSL tetramer, thereby sustaining cellular AMP and ATP production.

### TRAF3 restoration or AMP treatment partially rescue sarcopenia in Myo-cKO mice

**Figure 5.**
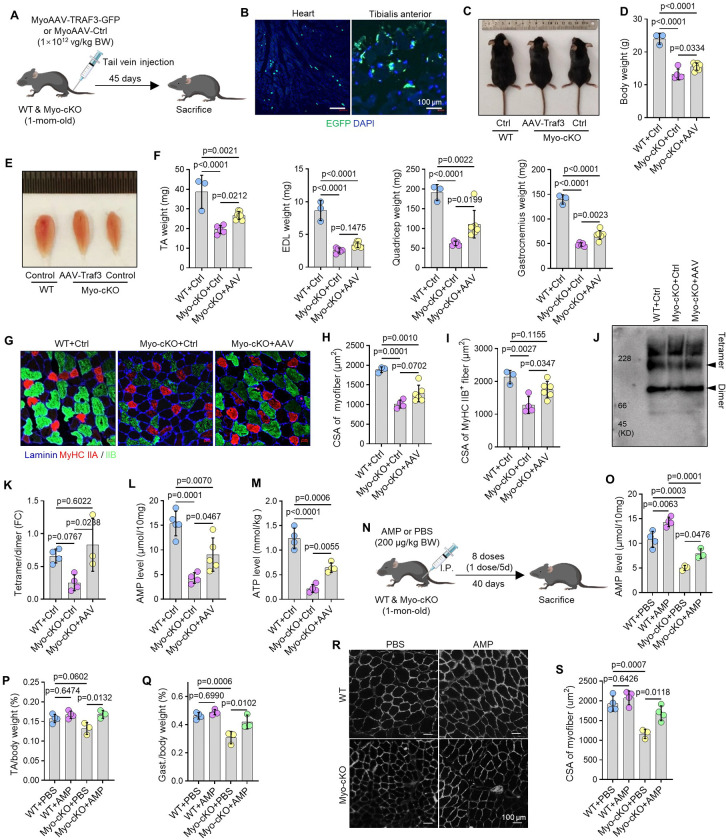
TRAF3 restoration or AMP treatment partially rescue sarcopenia in Myo-cKO mice. (A) Experimental design of Myo-cKO and WT mice being treated with MyoAAV-TRAF3-GFP and control (Ctrl) virus. (B) EGFP protein expression on cryosections of heart and TA muscle from Myo-cKO mice 45 d after MyoAAV-TRAF3-GFP injection. (C) Representative image of WT and Myo-cKO mice treated with MyoAAV-*Ctrl* or -*Traf3* viruses before sacrifice, and (D) their body weights. (E) Representative image of TA muscles from mice in (C), and (F) weights of TA, EDL, quadricep and gastrocnemius muscles. n=3 WT and 5 Myo-cKO mice treated with MyoAAV-*Ctrl* virus, and 7 Myo-cKO mice treated with MyoAAV-*Traf3* virus. (G) Representative images showing IF staining for MyHC IIA (red) and IIB (green) on cryosections of TA muscles from mice in (C), and (H) CSA of myofibers and (I) of type IIB fibers. n=3 WT and 4 Myo-cKO mice treated with MyoAAV-*Ctrl* virus, and 6 Myo-cKO mice treated with MyoAAV-*Traf3* virus. (J) Immunoblotting based on Native-PAGE of ADSL tetramer and dimer levels in skeletal muscles from WT and Myo-cKO mice treated with MyoAAV-*Ctrl* or -*Traf3* viruses, and (K) fold change of ADSL tetramer to dimer. n=4 WT and Myo-cKO mice treated with MyoAAV-*Ctrl* virus, and 3 Myo-cKO mice treated with MyoAAV-*Traf3* virus. (L) AMP levels in skeletal muscle lysates. n=5, 4, 5 mice for MyoAAV-*Ctrl*-treated WT and Myo-cKO mice, and MyoAAV-*Traf3*-treated Myo-cKO mice, respectively. (M) ATP levels in skeletal muscle lysates. n=4 mice/group. (N) Experimental design of PBS and AMP administration (I.P., 200 μg/kg body weight for 40 d) in Myo-cKO and WT mice. (O) Levels of AMP in skeletal muscle lysates. n=4 WT mice and 3 Myo-cKO mice treated with PBS or AMP. (P-Q) Weights of TA and gastrocnemius muscles, normalized to body weight, from mice in (M). n=4 WT mice and 3 Myo-cKO mice treated with PBS or AMP. (R) IF staining for laminin expression on cryosections of TA muscles from WT and Myo-cKO mice treated with PBS and AMP, and (S) CSA of myofibers of TA muscles. n=4 WT mice and 3 Myo-cKO mice treated with PBS or AMP. Analysis: one-way ANOVA with Tukey post analysis.

Having established a causal role for TRAF3 deficiency in age-related sarcopenia, we next asked if the sarcopenic phenotype was reversible. We performed muscle-specific TRAF3 restoration by administering an adeno-associated virus with muscle tropism^34^ (MyoAAV) expressing TRAF3 (MyoAAV-TRAF3-GFP) or a control vector (MyoAAV-Ctrl) to 1-mon-old Myo-cKO and WT mice via tail vein injection, with analysis conducted 45 days later (Fig. 5A). Immunofluorescence confirmed robust transgene expression in the tibialis anterior (TA) and heart muscles of treated Myo-cKO mice (Fig. 5B).

TRAF3 restoration yielded a modest but significant amelioration of the sarcopenic phenotype. MyoAAV-*Traf3*-treated Myo-cKO mice had a slight increase in body size and weight compared to MyoAAV-*Ctrl*-treated Myo-cKO littermates (Fig. 5C–D). Importantly, the mass of the TA, EDL, quadriceps, and gastrocnemius muscles was significantly increased, though mean values did not fully normalize to WT levels (Fig. 5E–F). Furthermore, the CSA of myofibers, particularly the atrophic Type IIB subset, was significantly larger, and Type IIA myofiber numbers were significantly higher in MyoAAV-*Traf3*-rescued than MyoAAV-*Ctrl*-treated Myo-cKO mice (Fig. 5G–I; Suppl. Fig. 5A-B). Mechanistically, TRAF3 restoration in Myo-cKO muscle significantly increased the ratio of the active ADSL tetramer to its inactive dimeric form (Fig. 5J–K). Concordantly, muscle AMP and ATP levels were significantly elevated in these mice (Fig. 5L–M).

To test directly if the metabolic deficit drives the sarcopenic phenotype, we administrated AMP to Myo-cKO mice (Fig. 5N). AMP treatment significantly increased skeletal muscle AMP levels in both Myo-cKO and WT mice (Fig. 5O). Strikingly, AMP supplementation significantly increased the body-weight-normalized mass of the TA and gastrocnemius muscles in Myo-cKO mice, but not in WT mice (Fig. 5P–Q). The CSA of TA myofibers was also significantly increased in AMP-treated Myo-cKO mice (Fig. 5R–S).

Collectively, these rescue experiments demonstrate that either restoring TRAF3 expression or bypassing its metabolic consequence via AMP supplementation can partially reverse the sarcopenic phenotype, confirming the functional importance of the TRAF3-ADSL-AMP axis.

### TGFβ1 induces TRAF3 ubiquitination and degradation in skeletal muscle during aging

**Figure 6.**
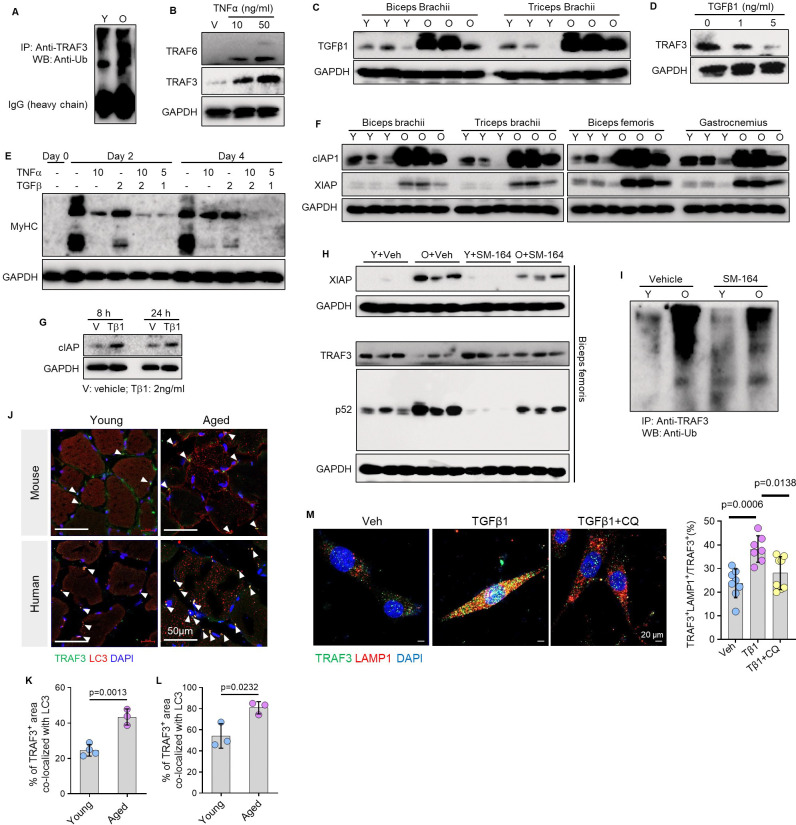
TGFβ1 induces TRAF3 ubiquitination and degradation in skeletal muscle during aging. (A) Protein lysates of skeletal muscles from young (Y; 3-mon-old) and old (O; 20-mon-old) mice pulled down using anti-TRAF3 Ab, and associated protein tested using anti-ubiquitin Ab by WB. (B) WB of TRAF6, TRAF3 and GAPDH in protein lysates of C2C12 myoblasts treated with increasing doses of TNFα. (C) Protein levels of TGFβ1 and GAPDH in lysates of biceps brachii and triceps brachii muscles from young (3-mon-old) and old (20-mon-old) mice. (D) WB of TRAF3 and GAPDH in protein lysates of C2C12 myoblasts treated with increasing doses of TGFβ1. (E) WB of MyHC and GAPDH in TNFα- and TGFβ1-treated C2C12 myoblasts. Units: ng/ml. (F) Protein levels of cIAP, XIAP and GAPDH in lysates of various muscles from young (3-mon-old) and old (20-mon-old) mice. (G) Protein levels of cIAP and GAPDH in C2C12 myoblasts treated with TGFβ1 (2 ng/ml) for 8 and 24 hr. (H) WB of XIAP, TRAF3, p52 and GAPDH in protein lysates of gastrocnemius muscles from young (2-mon-old) and old (20-mon-old) mice treated with the IAP inhibitor, SM-164 (I.P., 3 mg/kg/d, once/d for 1 mon). (I) Protein lysates of skeletal muscles from vehicle- and SM-164-treated young (3-mon-old) and old (20-mon-old) mice pulled down using anti-TRAF3 Ab, and associated protein tested using anti-ubiquitin Ab by WB. (J) IF staining for TRAF3 and LC3 expression on cross-cryosections of TA muscles from young and aged mice, and of gluteus maximus specimens from young and old humans. (K-L) Co-localization of TRAF3 with LC3 protein in skeletal muscles from young and old mice and humans. (M) Area of TRAF3^+^LAMP1^+^ in C2C12 myoblasts treated with TGFβ1 and vehicle plus MG132. Analysis: Student’s unpaired *t* test in (K-L), and one-way ANOVA with Tukey post analysis in (M).

We next sought to define the upstream mechanism responsible for TRAF3 loss in aged muscle. Analysis of TRAF3 ubiquitination revealed an age-dependent accumulation of ubiquitin-conjugated TRAF3 in TA muscles (Fig. 6A). While TNFα levels rise in aging muscle and stimulate TRAF6 expression^20^, treatment of C2C12 myoblasts with TNFα unexpectedly increased TRAF3 expression (Fig. 6B), suggesting that the age-related decline in TRAF3 occurs independently of TNFα signaling. In contrast, TGFβ1 protein levels were markedly elevated in aged biceps and triceps brachii muscles (Fig. 6C; Suppl. Fig. 6A-B). Given that primary myocytes from aged mice express TGFβ1 receptors I and II (Suppl. Fig. 6C), we treated myoblasts with TGFβ1 and observed a dose-dependent reduction in TRAF3 protein (Fig. 6D), suggesting a novel role for TGFβ1 in age-related sarcopenia. Interestingly, TGFβ1 acted synergistically with TNFα to promote myosin heave chain (MyHC) degradation, with the combination inducing greater loss than either cytokine alone (Fig. 6E).

We then investigated involvement of inhibitor of apoptosis proteins (IAPs), which mediate TRAF3 ubiquitination in age-related osteoporosis^33^. Levels of cIAP1/2 and XIAP were significantly higher in various skeletal muscles from old than from young mice (Fig. 6F; Suppl. Fig. 6D-G). Furthermore, TGFβ1 treatment increased cIAP protein levels in myoblasts (Fig. 6G), suggesting that TGFβ1 may promote IAP-mediated TRAF3 ubiquitination. To test this functionally, we treated old mice with the IAP inhibitor, SM-164, for one month. SM-164 treatment reduced XIAP levels, preserved TRAF3 protein, and decreased p52 levels in biceps femoris muscle compared to vehicle-treated controls (Fig. 6H; Suppl. Fig. 6H-J). This preservation of TRAF3 correlated with a reduction in its ubiquitination (Fig. 6I).

Immunofluorescence analysis revealed that TRAF3 accumulates within lysosomes in old mouse and human skeletal muscle (Fig. 6J–L). In vitro, TGFβ1 stimulation promoted TRAF3 localization to LAMP1-positive lysosomes in myoblasts, a process that was primarily inhibited by the lysosome inhibitor, chloroquine, (Fig. 6M). These data indicate that age-related, TGFβ1-driven TRAF3 degradation involves IAP-mediated ubiquitination and proceeds mainly through proteasomal pathways.

### Specific depletion of TGFβ1 in neutrophils prevented sarcopenia during aging

**Figure 7.**
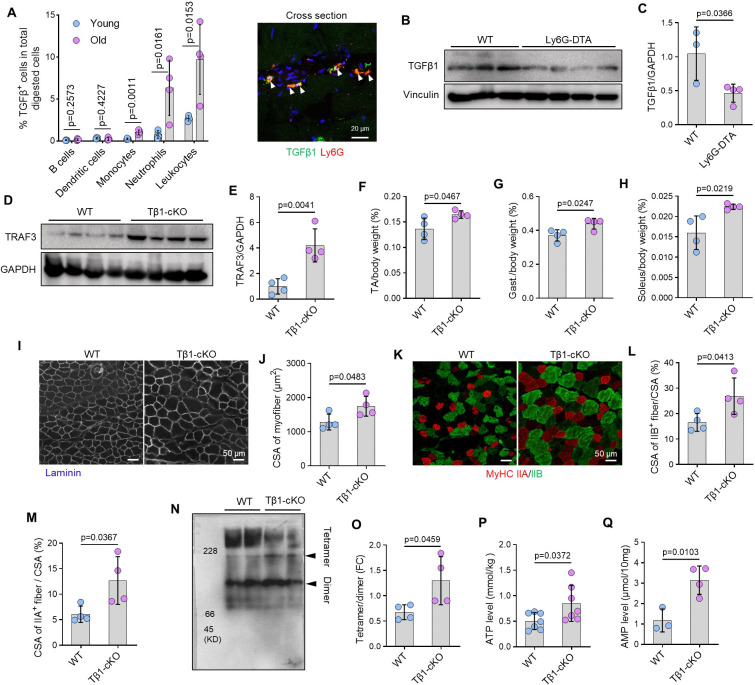
Specific depletion of TGFβ1 in neutrophils prevented sarcopenia during aging. (A) Detection and characterization of TGFβ1-expressing immune cells digested from skeletal muscles of young (3-mon-old) and old (20-mon-old) C57 mice (left panel), and detection of TGFβ1-expressing neutrophils in cross-section of frozen TA muscles from old (20-mon-old) C57 mice (right panel). (B) TGFβ1 and GAPDH protein levels in skeletal muscles from 18-mon-old Rosa26^DTA^ (WT) and Ly6G^CreER^Rosa26^DTA^ (Ly6G-DTA) mice tested by WB, and (C) relative levels of TGFβ1 normalized to GAPDH. n=3 WT and 4 Ly6G-DTA mice. (D) TRAF3 and GAPDH protein levels in skeletal muscles from 20-mon-old TGFβ1^fl/fl^ (WT) and Ly6G^CreER^TGFβ1^fl/fl^ (Tβ1-cKO) mice tested by WB, and (E) relative levels of TRAF3 normalized to GAPDH. n=4 mice per group. (F-H) Mass of TA, gastrocnemius, and soleus muscles, normalized to body weight, from 20-mon-old WT and Tβ1-cKO mice. n=4 mice/group. (I) IF staining for laminin expression in TA cryosections, and (J) CSA of myofibers of TA muscles from WT and Tβ1-cKO mice. n=4 mice/group. (K) Representative IF images of MyHC IIA (red) and IIB (green) in cryosections of TA muscles from 20-mon-old WT and Tβ1-cKO mice, and (L-M) CSA of type IIA and IIB fibers. n=4 mice/group. (N) Immunoblotting based on Native-PAGE of ADSL tetramer and dimer in protein lysates of skeletal muscle of 20-mon-old WT and Tβ1-cKO mice, and (O) relative levels of ADSL tetramer to dimer. n=4 samples/group. (P) ATP levels in skeletal muscle lysates. n=7 mice/group. (Q) AMP levels in skeletal muscle lysates from 20-mon-old WT and Tβ1-cKO mice. n=3 WT and 4 Tβ1-cKO mice. Analysis: Student’s unpaired *t* test.

To examine the cellular source of TGFβ1 in old muscle, we performed flow cytometric analysis. This revealed that neutrophils constitute the predominant TGFβ1-expressing immune cell population in old skeletal muscle (Fig. 7A; Suppl. Fig. 7A-C), which was confirmed by observing TGFβ1^+^Ly6G^+^ neutrophils in old skeletal muscle using immunofluorescence staining (Fig. 7A). Supporting their role as a key source, old mice with inducible neutrophil-specific deletion (Ly6G^CreER^Rosa26^DTA^; referred to as Ly6G-DTA) had significantly reduced skeletal muscle TGFβ1 levels compared to old WT controls (Fig. 7B–C).

To establish a direct causal link between neutrophil-derived TGFβ1 and sarcopenia, we generated old mice with a neutrophil-specific knockout of *Tgfβ1* (Ly6G^CreER^Tgfβ1^fl/fl^, referred to as Tβ1-cKO). Strikingly, skeletal muscle from old Tβ1-cKO mice had significantly higher TRAF3 protein levels than age-matched Tgfβ1^fl/fl^ (WT) controls (Fig. 7D–E). This preservation of TRAF3 was functionally consequential: Tβ1-cKO mice were protected from age-related muscle wasting, having increased body-weight-normalized mass of the TA, soleus, and gastrocnemius muscles (Fig. 7F–H). Myofiber atrophy was also ameliorated, with Tβ1-cKO mice showing significantly larger myofiber CSA, particularly in Type IIA and IIB myofibers, compared to age-matched WT controls (Fig. 7I–M).

Mechanistically, the protection observed in Tβ1-cKO mice mirrored the effects of direct TRAF3 restoration. Muscles from old Tβ1-cKO had a significantly increased ADSL tetramer-to-dimer ratio (Fig. 7N–O) and elevated levels of ATP and AMP (Fig. 7P–Q). Thus, neutrophil-derived TGFβ1 is a critical upstream driver of TRAF3 degradation and energy deficiency in age-related sarcopenia.

## Discussion

**Figure 8.**
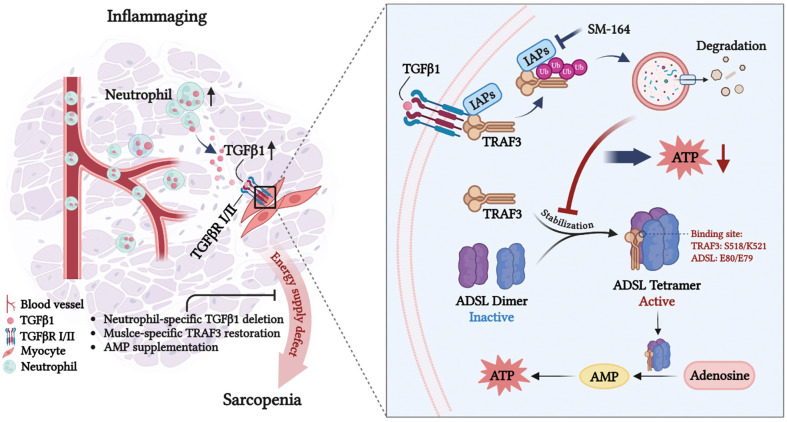
Schematic diagram summarizes the key role of TRAF3 in age-related sarcopenia. The skeletal muscle niche in aged mice is characterized by a state of chronic, low-grade inflammation that drives neutrophil recruitment and increased TGFβ1 secretion by neutrophils. Upon engaging myocyte surface receptors, increased TGFβ activates a signaling cascade that culminates in the ubiquitination and lysosomal degradation of TRAF3. Within myocytes, TRAF3 serves a critical scaffolding function; its binding via residues S518/K521 to ADSL (E80/E79) is essential for maintaining the stability of the catalytically competent ADSL tetramer. TRAF3 depletion destabilizes this complex, causing a precipitous decline in ADSL activity. This impairs purine nucleotide metabolism, creating an energy shortfall through reduced AMP/ATP generation. The resultant bioenergetic deficit translates directly into the functional decline of skeletal muscle, clinically manifested by progressive fiber atrophy, loss of contractile strength, and a failure of regenerative responses, which collectively define the sarcopenic phenotype. This figure created with BioRender.com.

Low-level chronic inflammation (LLCI) is increasingly recognized as a key contributor to various age-related pathologies, including sarcopenia^35^. This study unveils a novel inflammation-metabolism axis in age-related sarcopenia, linking neutrophil-derived TGFβ1 to TRAF3 degradation, impaired ADSL activity, and resultant energy supply defects to age-related muscle wasting. Our central finding is that under homeostatic conditions in young skeletal muscle, TRAF3 directly binds to adenylosuccinate lyase (ADSL), an essential enzyme in de novo AMP synthesis, to stabilize its active tetrameric form, thus ensuring efficient AMP and ATP biosynthesis critical for muscle energy demands. During aging, the accumulation of TGFβ1-expressing neutrophils in skeletal muscles leads to TGFβ1-induced increased IAP-mediated TRAF3 degradation and a significant reduction in active ADSL tetramers in myocytes, ultimately impairing energy supply and driving sarcopenia (Fig. 8). Therefore, this study identifies a novel, NF-κB-independent role for TRAF3 to prevent inflammation-induced energy supply defects in young/adult mice and maintain muscle health.

TRAF proteins are crucial intracellular signaling adaptors that mediate signals from TNF receptor superfamily and Toll-like receptor family members^36^. While previous studies have shown that TRAF6 mediates TNFα-induced muscle atrophy^20^ and TRAF3 acts as a negative regulator of non-canonical NF-κB signaling^37^, our data reveal a distinct role for TRAF3 in maintaining muscle health through metabolic regulation. We observed that TRAF3 protein levels are significantly reduced in old human and mouse skeletal muscles, in both myocytes and satellite cells. This decline appears to be a direct consequence of age-associated serum factors, as myoblasts exposed to old mouse serum had reduced TRAF3 protein levels compared to those treated with young serum. Myocyte-specific (Myo-cKO) and satellite cell-specific (Pax7-cKO) TRAF3 knockout in mice resulted in early-onset sarcopenia and impaired myofiber regeneration following injury with BaCl_2_, highlighting TRAF3’s essential role. The less severe sarcopenic phenotype in Pax7-cKO mice might be due to the lower efficiency of tamoxifen-inducible CreER in driving gene knockout.

Constitutive NF-κB activation is one of the key molecular drivers of LLCI during aging^38^. Canonical NF-κB signaling is triggered by inflammatory cytokines, such as TNF-α and IL-1β, while non-canonical NF-κB signaling is activated by BAFF, CD40L, lymphotoxin, RANKL and TGFβ1^39, 40^. TRAF3 is a known negative regulator of non-canonical NF-κB signaling^41^, and its degradation leads to release of NF-κB inducing kinase (NIK) and nuclear translocation of the non-canonical NF-κB proteins, p52 and RelB^27^. In this study, despite the observed increase in p52 and RelB in TRAF3-deficient mice, our results indicate that this pathway is not the primary driver of sarcopenia in this context. Global knockout of p52 and RelB genes did not increase muscle mass in mice, and pharmacologic inhibition of NF-κB with PDTC administration did not reverse muscle loss in TRAF3-deficient mice. These findings support an NF-κB-independent mechanism for TRAF3’s role in sarcopenia.

Homeostatic energy supply plays a critical role in maintaining skeletal muscle health^31^. A correlation between inflammaging and energy supply defects in age-related pathologies has been largely undocumented. Metabolomic and proteomic analyses revealed that TRAF3 deficiency primarily impacts the “Metabolism” pathway, leading to significantly lower levels of AMP and ATP in skeletal muscle. We identified a direct physical interaction between TRAF3 and ADSL, a key enzyme in purine biosynthesis. Specifically, TRAF3 binding at a specific interface (Ser518/Lys521 of TRAF3 with Glu80/Glu79 of ADSL) is critical for stabilizing the active ADSL tetramer to ensure stable synthesis of AMP and ATP. Sufficient supply of ATP is necessary for nucleotide metabolism, such as DNA and RNA synthesis, since ATP not only provides biological energy for initiating DNA transcription and elongation, but also provides its substrate, AMP, as the building blocks to support RNA synthesis^42^. Additionally, insufficient nucleotide synthesis can inhibit the synthesis of proteins, such as myosin, vital for skeletal muscle maintenance and repair^43, 44^. Therefore, this study pioneers the understanding of a critical mechanism whereby TRAF3 degradation impairs energy supply in skeletal muscles during aging, resulting in sarcopenia.

Infiltration of inflammatory cells, including macrophages, neutrophils, and T cells, into skeletal muscle affects muscle mass and regenerative capicity^45, 46^. Macrophages are known to shift towards a pro-inflammatory state during aging and increase their expression of TNFα, IL-1β, and IL-6^47^. TGFβ1 is a pivotal regulatory cytokine in maintaining skeletal muscle homeostasis^48^, and it has both beneficial and detrimental roles, partially depending on its level and timing^49^. The physiological mechanism whereby TGFβ1 maintains the integrity of extracellular matrix^50^, the quiescence status of satellite cells^51^, and anti-inflammatory effects is by dampening immune cell activation^52^. However, the role and cellular source of TGFβ1 in sarcopenic muscles during aging has remained elusive. Our study demonstrates that an age-related increase in neutrophil-derived TGFβ1 promotes IAP-mediated ubiquitination and lysosomal degradation of TRAF3, leading to reduced ADSL activity and energy supply defects. Consistent with this, old Ly6G-DTA mice with neutrophil-specific deletion had lower TGFβ1 protein levels than WT controls, and neutrophil-specific TGFβ1 deletion prevented age-related sarcopenia in old mice (Fig. 7), indicating the significance of neutrophils as a cellular source of TGFβ1 and of their role in causing age-related sarcopenia.

The study has some limitations. The *in vivo* models provided a robust causal link, but the generalizability of these findings to all aspects of human sarcopenia requires further clinical investigation. Aged *p52* and *RelB* gene global knockout mice and muscle-specific NF-κB knockout mice would be useful models for better understanding the role of NF-κB in age-related sarcopenia. Additionally, while we focused on the NF-κB-independent mechanism, the synergistic effect of TGFβ1 and TNFα on myosin heavy chain (MyHC) degradation warrants further investigation into the complex interplay of inflammatory cytokines during aging.

In conclusion, inflammaging-induced TRAF3 degradation in myocytes and satellite cells is a critical, NF-κB-independent driver of age-related sarcopenia, primarily by impairing ADSL function and subsequent AMP/ATP biosynthesis. These findings highlight a novel inflammatory-metabolic mechanism in muscle aging and suggest that targeting the neutrophil-derived TGFβ1/TRAF3 axis or restoring energy supply could be effective therapeutic strategies for preventing age-related sarcopenia.

## Methods

### Human clinical samples

Our study protocol was approved by the Human Investigation and Medical Ethics Committee of Hebei Medical University (Approval ID: 2021059). Informed consent was obtained from all patients or their guardians. Gluteus maximus samples were collected from 4 young patients (18–35 years old; 1 male and 3 females) and 9 elderly patients (59–73 years old; 2 males and 7 females) undergoing surgery for treatment of osteonecrosis of the femoral head. These samples would otherwise have been routinely discarded during the surgical procedures.

### Animals

To generate myoblast-specific TRAF3 knockout mice (Myo-cKO), TRAF3^flox/flox^ mice (GemPharmatech; Strain ID: T016527) were crossed with MCK^Cre^ mice (Jackson Laboratory; Strain ID: 006475). Myo-cKO mice and their wild-type (TRAF3^flox/flox^) littermates were sacrificed at 2 months of age for analysis. For generation of satellite cell-specific TRAF3 deletion mice (Pax7-cKO), TRAF3^flox/flox^ mice were crossed with Pax7^CreERT2^ mice (Jackson Laboratory; Strain ID: 017763). Tamoxifen (Aladdin; Cata#: T137974) was administered intraperitoneally (75 mg/kg body weight) to animals older than 2 weeks old or subcutaneously to younger animals. Pax7-cKO and control littermates were analyzed at 3 months of age. Neutrophil-specific TGFβ1 knockout mice (Tβ1-cKO) were generated by crossing TGFβ1^flox/flox^ mice (Jackson Laboratory; Strain ID: 033001) with Ly6G^CreERT2^ mice (Shanghai Model Organisms Center; Cata#: NM-KI-200218). Neutrophil-specific deletion mice (Ly6G-DTA) were generated by crossing ROSA26^DTA^ mice (Jackson Laboratory; Strain ID: 009669) with Ly6G^CreERT2^ mice. These mice and their littermate controls were maintained until approximately 20 months of age, with tamoxifen administered intraperitoneally at 100 mg/kg body weight. Wild-type C57BL/6 mice were purchased from Beijing HFK Bio-Technology Co., Ltd and analyzed at 2–3 months (young) and 18 months (old) of age. Mice were randomized and grouped according to body weight for studies. All animal experiments were performed in accordance with institutional guidelines and approved by the Animal Care and Use Committee of Hebei Medical University (Approval ID: IACUC-Hebmu-P-2025227).

### Cell culture and transfection

The mouse myoblast cell line (C2C12) was purchased from the Stem Cell Bank, Chinese Academy of Sciences, (Cata#: SCSP-505). Cells were cultured in DMEM (Gibco; Cata#: C11995500BT) supplemented with 10% fetal bovine serum (FBS; Hycyte; Cata#: FBP-C520) and 1% penicillin/streptomycin (Pen/Strep) in a humidified incubator at 37 °C with 5% CO_2_. Prior to transfection, C2C12 cells were seeded in 6-well plates at a density of approximately 5 × 10^5^ cells per well and allowed to grow until reaching 70–90% confluency. Transfection was performed using a Lipofectamine^™^ 3000 Transfection Reagent (Invitrogen; Cata#: L3000001) according to the manufacturer’s protocol. For each well of a 6-well plate, 5 μL Lipofectamine^™^ 3000 was first diluted in 125 μL Opti-MEM. In a separate tube, 2.5 μg of the TRAF3-EGFP-HA or ADSL-EGFP-Flag plasmid were mixed with 5 μL of P3000^™^ reagent and 125 μL of Opti-MEM. The two mixtures were then combined and incubated at room temperature for 15 min before being added to the cells. Following transfection, cells were cultured for 2–4 d at 37°C, after which transfection efficiency was assessed by Western blot analysis using standard protocols.

### Skeletal muscle dissociation

Following dissection under sterile conditions, skeletal muscle tissues were immediately rinsed in Ham’s F-10 medium supplemented with 1% HEPES and 10% horse serum (Hyclone; Cata#: SH30074–03) to remove residual blood and non-muscle connective tissues were meticulously removed. The cleaned muscle was mechanically minced using sterile scissors for approximately 300 strokes until a homogenized slurry was achieved, which was then subjected to enzymatic digestion in 7.5 ml of 0.2% type II collagenase (Worthington; Cata#: LS004176) at 37°C for 60 min with gentle agitation. After digestion, the suspension was transferred to a 50 ml centrifuge tube, mixed with 15 ml of Ham’s F-10 medium containing 10% horse serum, and centrifuged at 210 × g (or 1,500 rpm) for 5 min. The resulting pellets underwent secondary mincing with a sterile scalpel for approximately 100 strokes before being resuspended in fresh medium and recentrifuged. For further dissociation, the tissue was incubated in 10 ml of Ham’s F-10 medium containing 0.01% type II collagenase and 0.04% dispase II (Sigma; Cata#: D4693) at 37°C with agitation for 1 hr. The digested suspension was then sequentially passed through a 16G needle 15 times and filtered through a 40 μm cell strainer. After centrifugation at 310 × *g* (or 1,800 rpm) for 10 min, erythrocytes were lysed, and the remaining cell pellet was resuspended in 1 ml of Ham’s F10 medium with 10% horse serum for cell counting.

### Myoblast sorting

For negative selection of myoblasts, freshly digested muscle cells were incubated with a cocktail of biotin-conjugated Abs against CD45 (Miltenyi; Cata#: 130–124-209), CD11b (Miltenyi; Cata#: 130–113-804), CD31 (Miltenyi; Cata#: 130–119-662), and Sca-1 (Miltenyi; Cata#: 130–126-949) at a concentration of 2 μL per 10^6^ cells. Abs were diluted in 100 μL of 0.5% BSA-PBS and incubated for 30 min at 4°C. Following washing with 0.5% BSA-PBS, cells were labeled with 5 μL of streptavidin MicroBeads (Miltenyi; Cata#: 130–048-101) per 10^6^ cells, diluted in 100 μL of 0.5% BSA-PBS, and incubated for an additional 20 min at 4°C. After magnetic separation using a MACS LS column (Miltenyi; Cata#: 130–042-401), the unlabeled (flow-through) fraction was collected as purified myoblasts.

### Myoblast culture and differentiation

Purified myoblasts (CD45^−^CD11b^−^CD31^−^Sca1^−^) were plated at a density of 2 × 10^4^ cells per well in 8-chamber slides (Thermo Fisher; Cata#: 177429) with 300 μL of growth medium consisting of DMEM supplemented with 10% horse serum, 1% penicillin/streptomycin (Pen/Strep), 1% HEPES, and 5 ng/ml fibroblast growth factor-basic (FGF-2). After 48 hr, the growth medium was gently removed and replaced with 300 μL of differentiation-promoting medium (Ham’s F10 supplemented with 20% FBS, 1% Pen/Strep, 1% HEPES, and 5 ng/ml FGF-2). The growth medium was refreshed every 48 hr for 4–6 d until cells reached approximately 70% confluence, as assessed by phase-contrast microscopy using low-power objectives (4× or 10×). To induce differentiation, the medium was switched to differentiation medium (DMEM containing 3% horse serum, 1% Pen/Strep, and 1% HEPES) for 36 hr, with half-medium changes performed gently every 12 hr to maintain optimal differentiation conditions.

### Histologic staining and immunofluorescence

For general histologic analysis, tibialis anterior (TA) muscles were fixed in 4% paraformaldehyde (PFA) for 48 hr, processed through alcohols, embedded in paraffin, and sectioned. 4-μm-thick sections were stained with hematoxylin and eosin (H&E) using standard procedures. For immunofluorescence staining, 10-μm-thick frozen muscle sections, stored at −80°C, were retrieved and fixed with 4% PFA at room temperature for 2 min, followed by three 5-min washes in phosphate-buffered saline (PBS). Tissues were permeabilized with 0.3% Triton X-100 in PBS for 15 min at room temperature. After three additional PBS washes, non-specific binding was blocked sequentially: first incubated with 10% normal goat serum in 0.3% Triton X-100/PBS for 30 min at room temperature, and then with 3% affinity-purified Fab fragments against mouse IgG (H+L) and IgM (Jackson ImmunoResearch; Cata#: 115–007-003/115–006-020) for 1 hr to block endogenous immunoglobulins. Sections were then incubated overnight at 4 °C with primary Abs against MyHC-IIA (DSHB; Cata#: SC-71; 1:40 dilution), MyHC-IIB (DSHB; Cata#: BF-F3; 1:40 dilution), and laminin (Sigma-Aldrich; Cata#: L9393; 1:1000 dilution). The following day, sections were equilibrated to room temperature for 40 min, washed 3 times with PBST (0.5% Tween-20 in PBS), and incubated with appropriate secondary Abs for 1 hr at room temperature. After final washes, sections were mounted using Vectashield medium containing DAPI (Vector Laboratories; Cata#: H-1200) and imaged with a Zeiss LSM900 Airyscan2 inverted microscope (Thornwood, NY, USA).

### Immunoblotting

Protein lysates were extracted from freshly collected C2C12 cells or skeletal muscle tissues using RIPA lysis buffer (Seven Biotech; Cata#: SW104) supplemented with a protease inhibitor cocktail (Thermo Fisher Scientific; Cata#: A32965). Lysates were sonicated and incubated on ice for 30 min, followed by centrifugation to obtain protein supernatants. Protein concentrations were determined using a BCA Protein Assay Kit (Seven Biotech; Cata#: SW101–02) according to the manufacturer’s instructions. The protein samples were mixed with 5 × loading buffer and denatured at 95°C for 8 min. Thirty μg of protein per sample were separated by 8–12% SDS-PAGE and transferred to polyvinylidene difluoride (PVDF) membranes (Millipore; Cata#: ISEQ00010) at 200 mA for 120 min in an ice bath. After transfer, the PVDF membrane was washed 3 times, 5 min each with TBST (0.1% tween-20 in TBS), followed by blocking with 5% non-fat milk in TBST at room temperature for 2 hr. The membranes were then washed again with TBST and incubated overnight at 4 °C with primary Abs. The next day, after extensive washing with TBST, membranes were incubated with horseradish peroxidase (HRP)-conjugated goat anti-mouse or anti-rabbit secondary Abs (1:5000 dilution) for 1 hr at room temperature. Protein bands were visualized using enhanced chemiluminescence (ECL) substrate (Thermo Fisher Scientific; Cata#: 34577) and imaged using a Bio-Rad ChemiDoc system.

### Co-immunoprecipitation (Co-IP)

C2C12 cells were transfected with TRAF3-HA or TRAF3-NLS-HA plasmids, and ADSL-Flag plasmids, and cultured for 48 hr. Following verification of transfection efficiency by WB, cells were harvested and lysed in RIPA buffer containing a protease inhibitor cocktail (Thermo Fisher Scientific; Cata#: A32965). After sonication and 30-min incubation on ice, lysates were centrifuged at 12,000 × g for 15 min at 4°C to collect supernatants. Protein concentration of cell lysates was determined using a BCA protein assay kit (Seven Biotech; Cata#: SW101–02) according to the manufacturer’s instructions. 500 μg of protein supernatant were incubated with 10 μg of mouse anti-HA-Tag monoclonal Ab (Abclone; Cata#: AE008) or anti-DYKDDDDK-Tag polyclonal Ab (Proteintech; Cata#: 20543–1-AP) overnight at 4°C. IP was performed using the Pierce^™^ Classic Magnetic IP/Co-IP Kit (Thermo Fisher; Cata#: 88804) according to the manufacturer’s protocol. Briefly, 25 μl of magnetic beads were pre-washed with lysis/wash buffer, added to the protein-Ab mixture, and incubated at room temperature for 1 hr with gentle mixing/rotation. Beads were then isolated using a magnetic rack, washed three times with IP lysis/wash buffer, and bound proteins were eluted with elution buffer. Eluates were denatured by boiling at 100°C for 10 min, resolved by SDS-PAGE, and analyzed by immunoblotting with the appropriate Abs.

### Native Page

Protein lysates were prepared from C2C12 cells or skeletal muscle tissues using NP-40 lysis buffer (Solarbio life sciences; Cata#: N8032) according to the manufacturer’s instructions. For native PAGE analysis, 40 μg of protein was mixed with 2 × Native Sample Loading Buffer (Sangon Biotech; Cata#: C506025) and separated on an 8% native gel prepared using a Native PAGE Preparation Kit (Sangon Biotech; Cata#: C631101). Proteins were subsequently transferred to PVDF membranes and probed with either anti-DYKDDDDK-Tag polyclonal Ab (Proteintech; Cata#: 20543–1-AP; 1:4000 dilution) or anti-ADSL polyclonal Ab (Proteintech; Cata#: 15264–1-AP; 1:500 dilution). Horseradish peroxidase (HRP)-conjugated goat anti-mouse or anti-rabbit secondary Abs (1:5000 dilution) were used for detection, and protein bands were visualized using enhanced chemiluminescence (ECL) and imaged with a Bio-Rad ChemiDoc system.

### Plasmids

TRAF3-EGFP-HA and Piezo1-EGFP-Flag plasmids were obtained from GeneChem (Shanghai, China; Cata#: GOSE0310769). An ADSL-EGFP-Flag expression plasmid was generated by replacing the mouse Piezo1 cDNA in the Piezo1-EGFP-Flag plasmid with mouse ADSL cDNA (NM_009634.6). To construct a TRAF3 nuclear localization sequence (NLS) mutant plasmid, site-directed mutagenesis was performed on the TRAF3-EGFP-HA plasmid using overlap extension PCR with PrimeSTAR Max DNA Polymerase (Takara; Cata#: R045A). The following primers were used: forward point mutation primer: 5’-CTA CGC TCG TCG TGC ACA GGA GGC CGT CAT GGG GAA GAC-3’; reverse point mutation primer: 5’-CTG TGC ACG ACG AGC GTA GTC ACG GAT CTT CCA GAT CAG CAC-3’; forward outboard primer: 5’-TAC AGA GTT CTT GAA GTG GTG GCC TAA CTA CGG CTA CA-3’; and reverse outboard primer: 5’-CCA AAT ACT GTT CTT CTA GTG TAG CCG TAG TTA GGC CA-3’. PCR products were assembled using In-Fusion^®^ Snap Assembly Master Mix (Takara; Cata#: 638947), resulting in a TRAF3 NLS sequence mutation from RDYKRRKQ to RDYARRAQ. Similarly, TRAF3-ADSL binding site mutants were generated by mutating key residues: TRAF3-Mut1-HA (R505A/T540A), TRAF3-Mut2-HA (S518A/K521A), and TRAF3-Mut3-HA (D509A/D514A/K521A). Plasmids expressing TRAFs (TRAF1–7) were constructed by replacing the TRAF3 cDNA in the TRAF3-EGFP-HA plasmid with the cDNA of others TRAFs. All constructs were verified by DNA sequencing before use.

### Flow cytometry

For detection of TRAF3 expression by myoblasts and satellite cells, 1 × 10^7^ cells from skeletal muscles of 2- and 18-mon-old C57Bl6/J mice were stained with a lineage-depletion cocktail containing 2μl of each biotin-conjugated anti-CD45, -CD11b, -CD31 and -Sca-1 Ab, and 1μl anti- ITGA7-APC antibody (Invitrogen; Cata#: MA5–23555) in 100 μl FACS buffer (PBS containing 2% FBS) at 4 ℃ for 30 min. Cells were then labeled with FITC-conjugated streptavidin for 20 min at 4°C, fixed and permeabilized using BD Cytofix/Cytoperm^™^ Fixation and Permeabilization Solution (BD Biosciences; Cata#: 554722) for 15 min at 4°C. Intracellular staining was performed with 1μl AF647-conjugated anti-TRAF3 Ab (Santa Cruz Biotechnology; Cata#: sc-6933) in 100 μl FACS buffer at 4°C for 30 min. For detection of TGFβ1-expressing immune cells, digested cells from the skeletal muscles of young and old mice were stained with TGFβ1 recombinant monoclonal Ab (Proteintech; Cata#: 81746–2-RR). Cells were washed twice and resuspended in DAPI-contained FACS buffer for further flow testing. Analysis was conducted on a BD FACSCelesta^™^ flow cytometer (BD Biosciences) and FlowJo software (version 10.8.1).

### RNA-seq studies

RNA sequencing was performed on TA and gastrocnemius muscles from WT and Myo-cKO mice as well as on gastrocnemius muscles from young and old mice. Subsequent procedures were conducted by LC-Bio Technology Co., Ltd. (Hangzhou, China) following standard protocols. Total RNA was isolated using TRIzol^®^ Reagent (Thermo Fisher; Cata#: 15596018), purified, and reverse-transcribed to cDNA using SuperScript^™^ II Reverse Transcriptase (Invitrogen; Cata#: 1896649). The resulting cDNA samples were used to synthesize U-labeled second-stranded DNAs with E. coli DNA polymerase I (NEB; Cata#: m0209), RNase- H (NEB; Cata#: m0297) and dUTP Solution (Thermo Fisher; Cata#: R0133), and subjected to sequencing on an Illumina NovaSeq^™^ 6000 platform. Bioinformatics analysis included quality control, alignment to the mouse genome, gene expression quantification, and differential gene expression analysis. Functional enrichment, sample clustering, and heatmap analyses were further conducted to derive insights from the data. To identify the age-associated inflammatory signature, transcriptomic data (GSE167186) were analyzed using DESeq2 (version 1.34.0) in the R environment (version 4.5).

### Metabolomics analysis

Metabolomic profiling was conducted on four biologically independent samples per group by Applied Protein Technology (Shanghai, China) following standard operating procedures. Raw data were processed with ProteoWizard and XCMS software for peak alignment, retention time correction, and peak area extraction. The data extracted via XCMS underwent a structured workflow consisting of metabolite identification, data preprocessing, and experimental data quality assessment. Subsequent data analysis included univariate and multivariate statistics, screening of differential metabolites, correlation analysis of altered metabolites, and KEGG pathway enrichment analysis.

### Proteomics analysis

Proteomics analysis was performed on two independent biological replicates per group. The sample preparation procedure involved protein extraction and digestion, SDS-PAGE analysis, and filter-aided proteome preparation (FASP) for enzymatic cleavage. Peptides were desalted using C18 Cartridges, lyophilized, and reconstituted in 40 μL of 0.1% formic acid. The peptide concentration was determined by measuring the absorbance at OD280. Following chromatographic fractionation, data were acquired via liquid chromatography-tandem mass spectrometry (LC-MS/MS) and searched against relevant protein databases. Subsequent bioinformatic analysis, performed on the identified proteins, primarily included the assessment of identification quantities, differential expression analysis, and functional annotation. The entire workflow, encompassing sample preparation, LC-MS/MS, and bioinformatics, was carried out by Applied Protein Technology (Shanghai, China) in accordance with their standard protocols.

### Skeletal muscle regeneration assay

To induce muscle injury, 2-mon-old Myo-cKO mice and WT littermates received intramuscular injections of 50 μL 1.25% barium chloride (BaCl_2_) solution into the TA muscle, and muscle tissues were collected 5 d post-injury. For Pax7-cKO mice and WT controls, tamoxifen was administrated at postnatal day 15 with subcutaneous injections (75 mg/kg/day) for 5 consecutive d and intraperitoneally injected again 2 mon later, followed by a TA injection of 50 μL 1.2% BaCl_2_ one mon after the second tamoxifen treatment. Harvested TA muscles were fixed in 4% PFA for 48 hr at 4°C, embedded in paraffin and sectioned. Serial sections (4-μm thick) were subjected to H&E) staining and immunohistochemical analysis using anti-myosin heavy chain (MyHC) Ab (R&D Systems; Cata#: MAB4470; 15μg/ml). For quantitative analysis, 3–6 randomly selected microscopic fields from H&E-stained sections per TA muscle were imaged to evaluate inflammatory/regenerative areas, while the cross-sectional area (CSA) of centrally nucleated fibers (CNFs) was measured using ImageJ software. In MyHC-immunostained sections, 3 representative fields from the regenerative region of each TA muscle were analyzed to assay the MyHC-positive area and the number of MyHC-positive centrally nucleated fibers per mm^2^.

### Detection for ATP and AMP

ATP content in skeletal muscle tissues and cellular samples was measured using an ATP assay kit (Solarbio life; Cata#: BC0300). Briefly, each g of freshly harvested skeletal muscle tissue was homogenized in 5–10 ml of ice-cold extraction buffer. Supernatants were collected after high-speed centrifugation at 10,000 *g* for 10 min at 4°C, transferred to new EP tubes, mixed with 500 μL chloroform by vigorous vortexing, and centrifuged at 10,000 *g* for 3 min at 4°C, and the collected supernatants were put on ice. For cells, C2C12 cells transfected with siTRAF3 were constructed by Hanbio Biotechnology (Shanghai, China) and cultured for 24 hr, followed by individual or co-transfection with plasmids encoding ADSL-Flag, TRAF3-HA and TRAF3-Mut1/2/3-HA and incubation for additional 24 hr. 5~10 ml of extraction solution were added to each 10^6^ cells, and the samples were homogenized, sonicated, and centrifuged at 10,000 *g* at 4℃ for 10 min. Supernatants were collected, mixed with 500 μL chloroform, and centrifuged at 10,000 *g* at 4℃ for 3 min, and the final supernatants were harvested and put on ice for subsequent ATP detection according to the manufacturer’s instructions. AMP content was measured using an AMP assay kit (Abcam; Cata#: ab273275). Cell and tissue samples were processed according to the manufacturer’s instructions, with absorbance measured at 570 nm to calculate AMP content.

### Virus preparation and injection

For *in vivo* gene delivery, we employed MyoAAV, a novel AAV capsid variant containing an RGD motif that has enhanced muscle tropism through integrin-mediated transduction, as previously described^34^. MyoAAV Virus Packaging and Concentration service was provided by Genomics Technology Co., Ltd (Shanghai, China). Three groups of mice: WT mice injected with PBS, Myo-cKO mice injected with PBS or MyoAAV were used in this study. The MyoAAV virus was injected into the tail vein of 1-mon-old Myo-cKO and WT mice at a dosage of 10^12^ vg/kg. Tissues were collected 45 d post-injection for analysis. IF staining was performed to assess the specificity of viral expression across different tissues.

### Grip strength test

Muscle strength was assessed using a dynamometer (KEW BASIS; KW-ZL-2). Each mouse was held by the tail and pulled backward until it released the handle. This test was performed 3 times following a 5-min rest period, and the peak grip force from 2 trials was recorded for analysis.

### Statistical analysis

All samples in this paper were randomly grouped, data collection was randomized, and sample size was not predetermined using statistical methods. Unless otherwise stated, all experiments were performed with at least 3 independent biological replicates, and data are presented as mean ± SD. All experimental data were statistically analyzed using GraphPad Prism (v.8.2.1) software. Two-group comparisons were analyzed by two-tailed Student’s *t* test, while multiple groups were analyzed using two-way ANOVA with multiple comparison test. The assumption of a normal distribution was made but not formally tested. A p value < 0.05 was considered statistically significant.

## Supplementary Material

Supplementary Files

This is a list of supplementary files associated with this preprint. Click to download.

• Suppl.Figures01252026.pdf

## Data Availability

All relevant data are available from the authors upon reasonable request.
